# Advancement of Nonwoven Fabrics in Personal Protective Equipment

**DOI:** 10.3390/ma16113964

**Published:** 2023-05-25

**Authors:** Dhanya Venkataraman, Elnaz Shabani, Jay H. Park

**Affiliations:** 1 Department of Biomedical and Biotechnology, University of Massachusetts Lowell, Lowell, MA 01854, USA; dhanya_venkataraman@student.uml.edu; 2 Department of Plastics Engineering, University of Massachusetts Lowell, Lowell, MA 01854, USA; eshaban@alumni.ncsu.edu

**Keywords:** nonwovens, personalized protective equipment, fibers, filtration, medical, protection

## Abstract

While nonwoven fabrics have existed for several decades, their usage in personal protective equipment (PPE) has been met with a rapid surge of demands, in part due to the recent COVID-19 pandemic. This review aims to critically examine the current state of nonwoven PPE fabrics by exploring (i) the material constituents and processing steps to produce fibers and bond them, and (ii) how each fabric layer is integrated into a textile, and how the assembled textiles are used as PPE. Firstly, filament fibers are manufactured via dry, wet, and polymer-laid fiber spinning methods. Then the fibers are bonded via chemical, thermal, and mechanical means. Emergent nonwoven processes such as electrospinning and centrifugal spinning to produce unique ultrafine nanofibers are discussed. Nonwoven PPE applications are categorized as filters, medical usage, and protective garments. The role of each nonwoven layer, its role, and textile integration are discussed. Finally, the challenges stemming from the single-use nature of nonwoven PPEs are discussed, especially in the context of growing concerns over sustainability. Then, emerging solutions to address sustainability issues with material and processing innovations are explored.

## 1. Introduction

Nonwoven fabrics are made of individual fibers, rather than yarns, that are tangled, bonded, or felted together. These are long (filament) or short (staple) fibers which are bonded together by heat (low melt fibers used and heat melts the fibers together), chemical (fibers are chemically bonded to each other), or mechanical treatments (fiber entanglement) [1,2]. While not woven or knit, nonwovens provide a feel of fabrics with random yet tortuous structures [3,4].

Nonwoven fabrics are typically used in applications that, namely, do not require apparel construction. Naturally, the production time is less as the manufacturing steps involved are fewer compared with woven or knit, thereby costing less [1,2]; the industrial production rate is typically 100 to 400 yards per minute. Owing to its cost effectiveness, nonwovens are more ideal for disposable products such as wipes, feminine hygiene, diapers, and others [2]. These nonwovens can be suitable for a variety of applications such as aerospace applications, acoustic/thermal insulation, fire retardant materials, industrial filters, puncture, cut-resistant materials, synthetic and composite materials for industrial uses, welding protection, wall coverings, truck liners, and vehicle seats [5].

In the current review, we aim to examine the state of nonwoven at two levels: (i) traditional and emerging production methods, and (ii) nonwoven applications. Given the timely importance of personalized protective equipment (PPE) due to the recent pandemic, we present nonwoven applications specifically for PPE across multiple sectors. The PPE applications, broadly, are categorized by filtration, medical, and protection, with a focus on their materials, assembly, and the functionalities of each layer. The state of the PPE nonwovens and challenges are discussed, especially in the context of growing concerns over the environment and supply chains, i.e., sustainability. Lastly, the outlook of nonwoven PPEs to address these challenges in the recent years will be discussed.

## 2. Fabrication of Nonwoven Textiles

**Figure 1:**
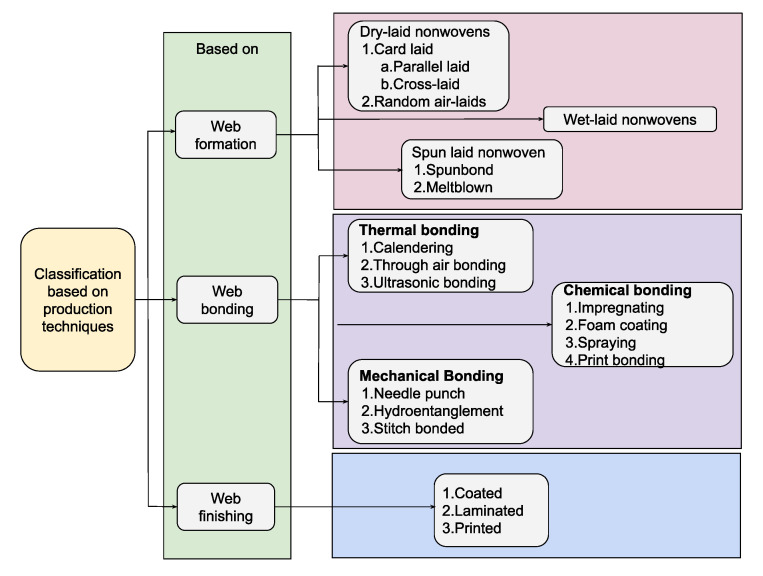
Classification of Nonwovens.

Nonwovens can be classified based on the raw materials used, the method of production, their properties, and the applications it would be used for [6]. Nonwoven manufacturing involves these steps: continuous filament and/or staple fiber fabrication, web formation, web bonding, and web converting and finishing [7]. Figure 1 shows the classification of nonwovens based on how they are manufactured. The following sections will briefly discuss nonwoven manufacturing processes from web formation to web bonding and finishing.

### 2.1. Nonwoven Web Formation

Nonwoven webs are manufactured by two approaches. The first method entails bundling staple fibers together, which are then transformed into a nonwoven web through dry, wet, or air-laid techniques. On the other hand, the second method involves the direct fabrication of nonwoven webs from polymer resins, i.e., polymer laid. In the following subsections, we will provide a detailed overview of each of these methods.

#### 2.1.1. Dry, Wet, and Air-Laid Web

**Figure 2:**
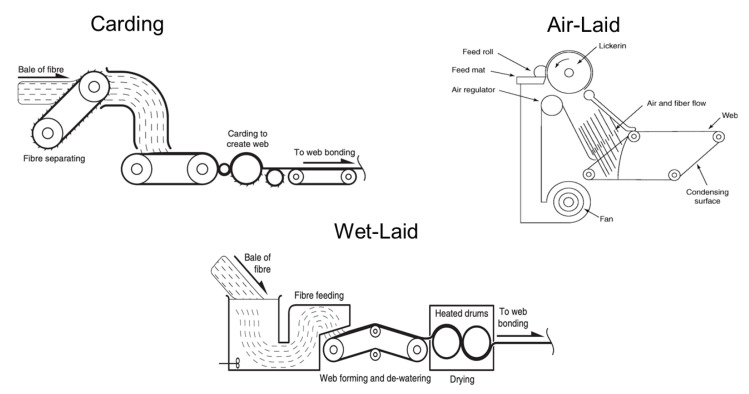
Post-fiber nonwoven formation processes [8].

Typically, continuous filament fibers are manufactured via wet, melt, or dry spinning. These filament fibers are then bundled as tows and cut into desired lengths. The chopped fibers are referred to as staple fibers, characterized by limited, finite, small length fibers [8]. Based on the specific end use, tows can be cut into staple lengths or flocks [8,9,10,11,12,13,14]. These fabrics can be constructed in a wide variety of specific masses and textures. Staple fabrics can accept dyes well, giving a natural and subtle look with the colors, although many factors have lessened the appeal for these fibers as the process is expensive and decreased demand only exacerbates it further [15,16,17,18]. The major issue with these staple yarns are pilling and fuzzing, as they are made of shorter fibers that are twisted together, which sheds loose filaments and necessitates extra maintenance and cleaning [15,16,17,18]. The staple fibers are arranged to loosely hold structures as webs, batts, or sheets [7,8,9,11,19,20,21]; these structures are porous due to the tortuous path between the fibers, which is a geometrical characteristic owing to the web formation process. The fiber materials are deposited on a dry, wet, or air-laid forming or conveying surface (see Figure 2 for an overview schematic of dry, web, and air-laid web formation processes).

In brief, dry-laid nonwovens are formed by carding followed by web lappers converting fibers into a web structure; the web of fibers is laid in a drum and hot air is injected to bond the fibers together [11,21,22,23]. In carding, staple fibers are passed through a series of rotating drums covered in fine wires to comb them into a parallel or random arrangement, creating a “batt” which are fed continuously onto a conveyor belt to form a web. Air-laying entails separating fibers that are dispersed into a fast-moving air stream and transferred to a moving screen by means of pressure or vacuum, deposited in the form of a web [11,19]. Air-laid nonwovens find applications in absorbency such as diapers, napkins, wipes, tablecloths, disposable medical gowns, wound care dressings, feminine hygiene products, filtration media, and insulation [21,23,24]. Lastly, the wet-laid process is analogous to papermaking; web is formed by being dispersed in a liquid or water which is then subsequently layered. Fibers that are suitable for the process disperse in the aqueous medium and swell; then, the mixing vats are sent to a head box from which they are fed continuously to a web-laying machine [11,25]. The web is then drained and consolidated by pressing between rollers and drying. Fabrics made from the wet-laid process have paper-like qualities and are used as interlinings, tea bags, aprons, bed linens, gloves, surgical gauze, napkins, fire-retardant protective apparel, disposable medical products, glass fiber roofing substrate, and insulation materials [8,26,27,28].

#### 2.1.2. Polymer-Laid Web Formation

**Figure 3:**
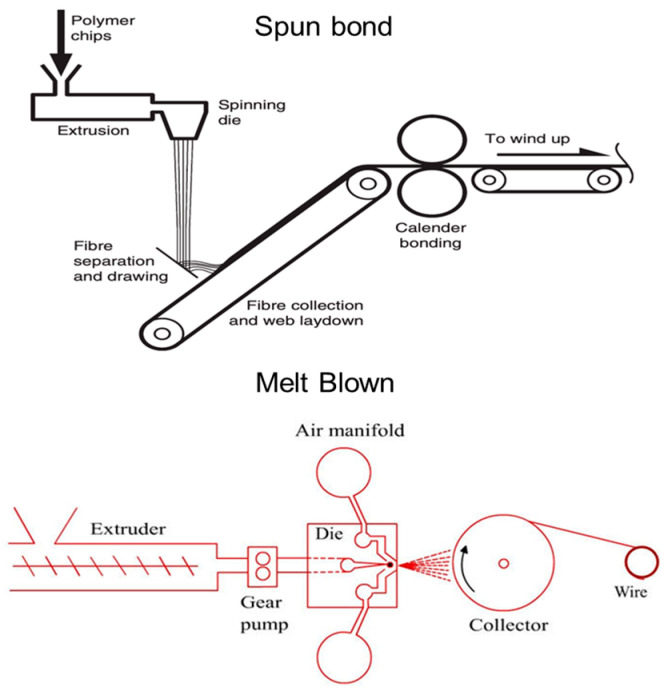
Schematics of two polymer-laid methods (spunbond [8] and meltblowing [34]).

Polymer-laid web formation is a process to convert polymer resin directly into a nonwoven web. Fabrics are produced by an extrusion spinning process, and filaments are directly collected to form a web instead of forming yarns as in conventional spinning. Most notably, post-fiber processing steps such as carding and cross-lapping are eliminated which result in higher production and the lowest cost of web formation [7,29]. The two traditional and dominant processes are spunbond and meltblown (see Figure 3); although the principles are similar, the processes entail distinct mechanisms [11,29].

Spunbond or spun laid nonwovens are made in one continuous process. The manufacturing steps entail forming filaments via an extruder, spinning filaments, drawing, depositing filaments onto a collecting belt, and bonding fibers by mechanical or thermal methods [11,30]. These continuous filaments are stretched, rapidly cooled, and quenched before depositing on a conveyor belt to form a uniform web [31]. Due to attenuation of the filaments, spunbond nonwovens have an increased strength compared with carding. Most fiber-forming thermoplastic polymers can be processed by spunbonding [7,21]. Spunbonds are used in a wide range of applications:

- Automotive—seat covers, interior door panels, trunk liners, etc.
- Civil engineering—roofing, erosion control, canal and reservoir lining protection, revetment protection, geosynthetics, railway bed stabilization.
- Packaging, geotextiles.
- Hygiene and medical—sanitary napkins, disposable operating gowns, personal protective equipment (PPE) [7,8,11,19,21,22,23,29,31,32,33].

Meltblown, like spunbond, begins with extruding a polymer melt through a linear die with several hundred small orifices. Instead of quenching the filaments, they are decreased by hot streams keeping them in a partially molten state leaving the spinneret [31,34,35,36]. This leads to thinner filaments with a lower tensile strength and finer diameter fibers (5–10 μm). High velocity air or another appropriate force rapidly attenuates the filaments that are blown onto a conveyor belt to form fine diameter fibers (1–5 μm) [7,8,22]. Meltblown fabrics have a high surface area used for enhanced filtration efficiency, excellent barrier properties, good insulation, and good wicking action. A large surface area enables air to be trapped through drag force on air convection forces through the fabrics, which is used in outdoor clothing products [13,14,15,16]. The difference between spunbond and meltblown is that the latter uses finer spinnerets combined with high temperature air to weaken the filaments to break during their formation. They are shorter in length compared with the continuous filaments of the spunbond process, and meltblown requires polymers with a lower melt viscosity [8,11,17,21]. The initial investment for spunbond is three to four times more than meltblown; however, meltblown consumes more energy than spunbond as it uses compressed hot air. Meltblown nonwoven is more expensive than spunbond nonwoven [34,35]. General meltblown characteristics and their corresponding applications are as follows:

- Enhanced filtration efficiency—air filters; food and beverage filtration; surgical mask and respiratory filtration; water, gaseous, and liquid filtration.
- Excellent barrier properties—used in insulation applications.
- Good wicking action—industrial wipes, oil sorbent pads and booms.
- Medical and hygiene—face masks, PP gowns, N95 mask filter fabrics, sanitary napkins, diapers, wipes, sterilization wraps, drape markets, and meltblown in masks and filters.
- Packaging—warm filling materials, filtering materials, silica gel bags, tea bag fabrics, and filter papers.
- Oil adsorbent—absorb oil spills on water surfaces [7,21,22,31,34,35].

#### 2.1.3. Emerging Polymer-Laid Web Technology

While meltblown and spunbond have dominated the landscape of polymer-laid nonwovens, emergent technologies that can produce ultrafine nonwoven fibers (*d* ≈ 0.1–1.0 µm) have come to the surface in last 30 years. These two nanofiber-forming technologies are electrospinning and centrifugal spinning [37,38].

**Figure 4:**
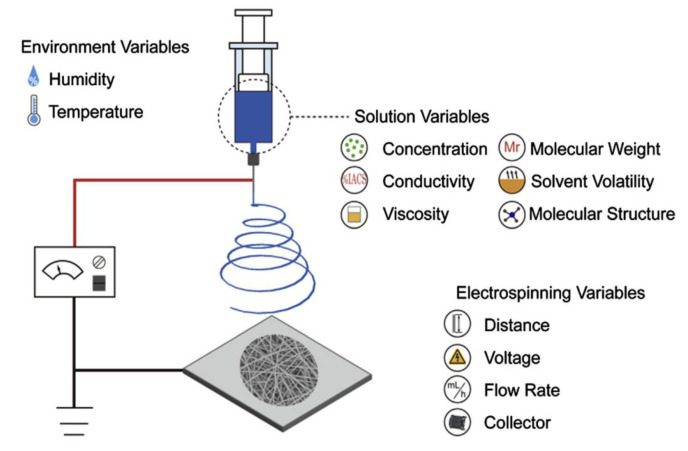
A schematic of electrospinning setup and mechanism [46].

Electrospinning is a simple, non-mechanical technique for fabricating nanofibers from polymer solutions by applying an electrical force. A single nozzle electrospinning system consists of a syringe, a metal nozzle, a power supply, and a collector (see Figure 4) [39,40,41,42,43,44]. In this process, a high voltage is applied between the syringe containing the spinning solution with a metal needle and the collector which draws the fibers to it. On reaching a critical value by the voltage, the electrically charged solution generates a conical droplet, a Taylor cone from which a liquid jet is formed, and the fibers are extracted [39,40,44,45,46,47]. This electrically charged jet undergoes a stretching and elongation through which the fiber diameters decrease from μm to nm. The spun nanofibers are collected and accumulated on the grounded collector [48]. Depending on the fiber collection, they become randomly arranged nonwovens, highly aligned nanofibers, or even yarns. Numerous interacting parameters influence the process and fiber formation, which include the polymer molecular weight, the solution concentration, the voltage, the surface tension, the solution flow rate, the nozzle diameter, the nozzle–collector distance, the conductivity, the rheological behavior of the polymer fluid, and the motion of the collector [39,40,41,43,44,45,46,47]. The drawbacks of this process are electrical hazard due to high voltage and low productivity per nozzle which can be scaled-up via a multi-nozzle approach; however, the process still linearly scales with the number of nozzles used [41,44,45,46,47].

**Figure 5:**
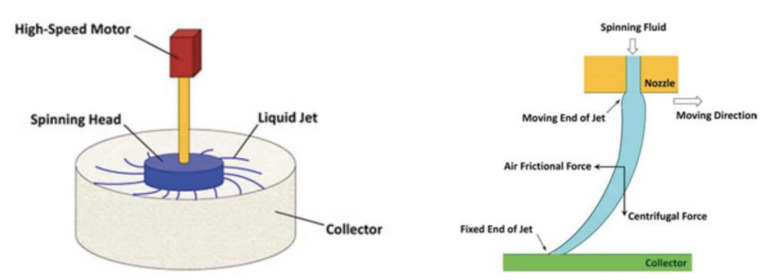
A schematic of centrifugal spinning setup and mechanism [40].

For centrifugal spinning, a polymer solution or melt is injected into the spinneret with multiple nozzles and is connected to a motor. The liquid jets are formed at the nozzle tips of the spinning head when the centrifugal force overcomes the surface tension of the spinning fluid (see Figure 5) [40]. The liquid jets come out of the nozzle tip, and the centrifugal force and air frictional force can elongate and solidify liquid jets into fibers with solvent evaporation. Rotational speed and solution concentration play a major role in forming continuous fibers, preventing jet breakup, and bead formation [40,41]. The stretched jets are deposited on the surface of the collector forming a nonwoven mat of nanofibers. It has a high production rate, with good safety and a low cost for industrial production [41]. There are different types of collectors being used, such as (i) the gravity-assisted nanofiber nonwoven collector, which collects fibers by using gravity, (ii) the suction force-assisted collector, in which the suction force draws the fibers and deposits them on the collector, (iii) the air jet-assisted collector, in which a jet of air flows to push the spun fibers to the collector to be deposited as nonwoven mats, and (iv) the water bath-assisted collector which collects the nanofiber yarns using a water bath and rotating roller [41].

**Figure 6:**
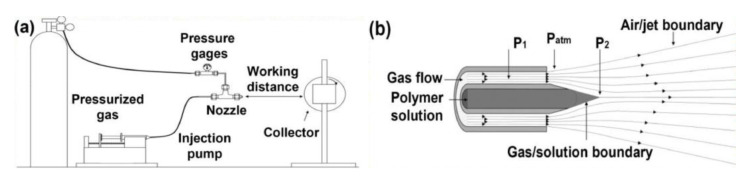
A schematic of solution blow spinning (**a**) setup and (**b**) mechanism [49].

Two additional emerging processes, solution blow spinning and flash spinning, have gained more interest recently. Solution blow spinning is a combination of electrospinning and meltblowing to prepare nanofibers. The process consists of compressed gas, an injection pump, a nozzle, and a collector, as seen in Figure 6 [49,50]. The process is based on high-speed draft and the Bernoulli principle, in which the polymers are dissolved in a volatile solvent to form a homogenous spinning solution. The solution is fed to the nozzle by an injection pump and a Taylor cone is formed at the nozzle along with the impact of a high-speed air flow. The jets are formed when the shear force generated by the high-speed air flow overcomes the surface tension of the solution [49,50]. The jet moves to the collector, the solvent gradually volatilizes, the fibers solidify, and, finally, the nanofibers are collected. Compared with electrospinning, it is safer as the typical voltage required is less. Compared with meltblown, a wider range of polymers can be used, as non-melt polymers, such as acrylics and cellulosic, can be processed [49,50].

**Figure 7:**
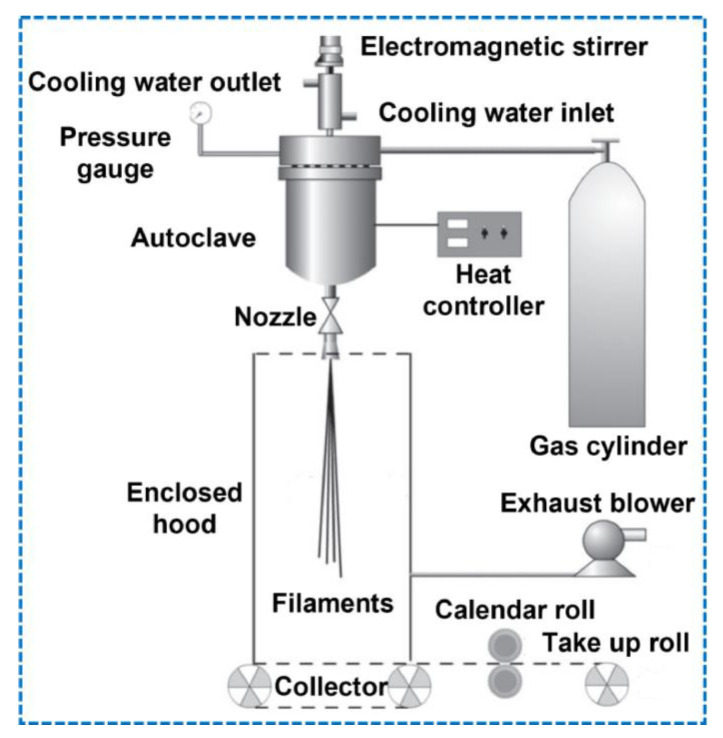
A schematic of Flash spinning apparatus [49].

Flash spinning produces micro/nanofibers or filamentary products from the spinning solutions which are based on the principles of phase separation and supersonic flow. It consists of four parts: an autoclave, a spinneret, a collector, and a high-speed airflow or electrostatic generator, as seen in Figure 7 [49]. To prepare a spinning solution, the polymers are dissolved in suitable solvents under high temperature and pressure. The spinning solution consists of polymer, a primary and secondary solvent, and additives. The solution then sprays out from the spinneret of the autoclave with rapid solvent evaporation due to a sudden pressure drop, after which the polymers are solidified and stretched to form ultrafine fibers by high-speed airflow. To prevent fiber entanglement, high-speed airflow and a high voltage electrostatic field are used [49]. Flash spun materials are ideal for use in air filtration and medical protective materials. Protective garments with flash spun fibers offer good comfort, with excellent mechanical properties and superior barrier resistance to pathogens and liquids [49]. Table 1 below summarizes the polymer-laid manufacturing methods discussed, noting their advantages, disadvantages, and applications.

### 2.2. Web Bonding

Bonding, which is needed in a nonwoven web to impart mechanical integrity, is when webs or layers of fibers, filaments, or yarns interlock by mechanical, chemical, or thermal means. The challenge is to take a formed web which is made from a soft, flexible, and porous material, lacking strength and durability, and to connect the fibers to give the characteristics of a finished nonwoven product [7,11,19]. The method and level of bonding determines the properties and strength of the fabric [8]. Sometimes, a combination of bonding is used to achieve high stiffness [19].

**Figure 8:**
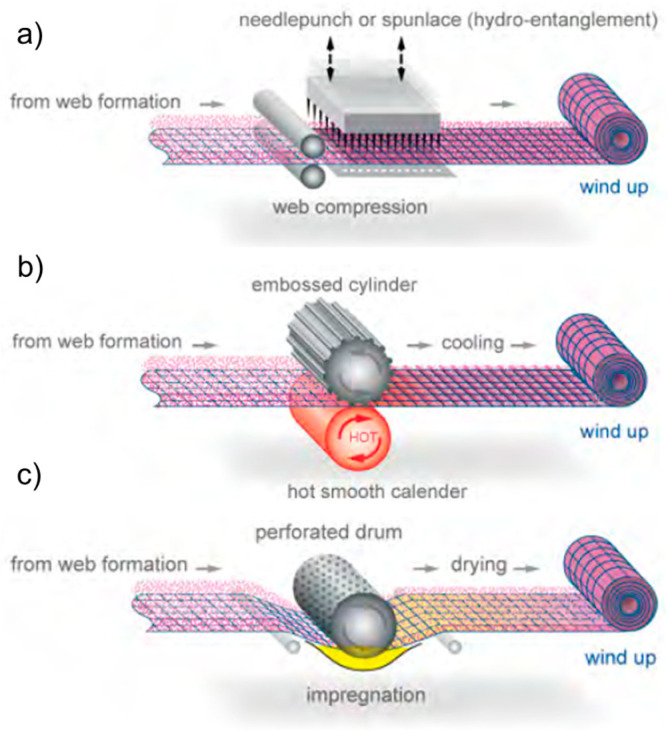
Schematics of nonwoven web-bonding processes driven by (**a**) mechanical, (**b**) thermal, and (**c**) chemical means [52].

- Mechanical bonding—needle punching, stitch bonding, hydrogen tangling (Figure 8a).
- Thermal bonding—through air, calendar bonding, or ultrasonic bonding (Figure 8b).
- Chemical bonding—spray, foam, print, impregnation (Figure 8c).

In mechanical bonding, the inherent characteristics of the fibers are unaffected as the effect of mechanical bonding on the absorbency of these fibers is minimal [14,39]. It involves the strengthening of the webs through the physical entanglement of fibers by barbed felting needles repeatedly passing in and out of the web [9]. The entanglement of the fibers causes two effects: it may restrict the inherent ability of the structure to swell, and the fabric may become more resilient and resist collapse when external pressure is applied, and these effects can significantly impact the capillary absorption of the fluid [15,16]. One example of mechanical bonding is needle punching, which interlocks the fiber webs by physically displacing the fibers from a near-horizontal to a near-vertical position [51]. Another mechanical bonding method includes stitch bonding, where fiber webs and/or yarns are bonded together by warp knitting and sewing techniques with continuous filaments to create a series of loops, interlocking the fiber webs to hold the web together [52]. Lastly, hydroentanglement entails entangling a nonwoven web of loose fibers, with fine, high-pressure jets of water to produce fabric surface-texturing and/or web consolidation [52]. As a water jet strikes the web, moving individual fibers away from the high points, voids are created in the web and the fibers intermingle [19,52]. When used for bonding, hydroentanglement repositions individual fibers into webs that result in frictional interlocking, while being used for surface texturing means the rearrangement of fibers into an open pattern [19]. The resulting fabric is comparatively smooth, flexible, and has a relatively strong structure due to a large amount of individual fiber entanglement.

Thermal bonding utilizes heat and pressure to soften and then fuse or weld fibers together, acting as an adhesive and staying below melting temperature. The steps involved in this process are heating the webs, forming a bond through the polymer and the fiber–fiber interface, and cooling the fibers [11]. When the fibers are heated to their glass transition temperature, the bonding fibers liquefy and surround the main fibers, which then bonds the web at fiber intersection points [8,53]. For example, the calendaring process is when the web of loose fibers passes between the nip of a pair of calendar rollers (heated or unheated) at a controllable speed to impart heat and pressure to the weld fibers [8,21]. The heat liquefies the thermoplastic fibers, compressed by pressure, and causes them to surround the other fibers in the web, locking them into place as they cool [8,54]. Embossing and engraving rollers bond the webs at specific points, mostly at the raised points of their surfaces. The fabrics produced are strong, relatively soft, lightweight, low-loft, and flexible [8,19]. They can be applied as a substrate for tufted carpets, geosynthetics, filtration media, protective/disposable clothing, coating substrates, and as hygiene covers [12,21,32]. Other examples include through-air bonding and ultrasonic bonding. The latter has been widely used to bond nonwoven layers using a high frequency oscillator or horn that generates sound waves which then convert to thermal energy to heat fiber webs [8,19].

Lastly, chemical bonding usually takes place in two stages—the application of a bonding agent and the triggering of the bonding agent using heat [8,11,19,21]. The binder is applied to the web in a solid form, such as powder, film, fiber, or foam, or in a liquid form, such as emulsion, dispersion, or solutions, which are predominantly water-based to bond the constituent elements or enhance adhesion [8,19]. The most frequently used chemical binder for nonwovens is waterborne latex—latex binders made from vinyl polymers such as polyvinyl acetate, polyvinyl chloride, styrene/butadiene resin, polyacrylic, or a combination of these [8,11,19,21]. It can also be used to color webs by adding pigments to the binder solutions [8]. The number of chemical binders used on webs can be between 5 and 60% by weight [11]. After the application of chemical binders, curing and drying by subjecting the subtracts to thermal bonding is necessary [21]. Representative chemical bonding processes include spray bonding, foam bonding, and print bonding. In spray bonding, the binder is applied to one or both sides of the web using high-pressure spray guns. In foam boding, a binder paste, or foam, is applied to the nonwovens by knife or scrapers, which are supported by air, a blanket, or rollers [8]. Lastly, print bonding entails chemical binders that are applied, or “printed”, onto the web using patterned rollers or rotary screens [8].

## 3. Nonwoven PPE Applications

Nonwoven fabrics are used in numerous applications which include hygiene, medical, filters, geotextiles, agriculture, personal, clothing, automotives, households, construction, industrial, footwear, army and soldiers’ protective clothing, and respirators. In this review, PPE applications from nonwovens are exclusively examined. Table 2 below lists PPE applications, associative products, the nonwoven methods used, and layer compositions. These applications can be broadly classified as filtration-based, medical applications, and workers’ protective garments.

### 3.1. Filtration

Nonwovens are used in all types of filtrations, air, water, oil, medical, gas, dust, odour, food, beverages, liquid, antimicrobial, biopharmaceutical, HVAC-industrial heating, ventilation, and air conditioning. Air filters can effectively capture dust, pollen, allergens, viruses, and other microscopic particles to protect, to reduce symptoms, and to breathe comfortably. Medical, healthcare-based ones are used to refrain from pathogens, bacteria, or viruses that would contaminate the sterile environment. Oil-based filters are used in vehicles, to remove any impurities from the engine oil, hydraulic oil, transmission oil, and lubricating oil. The following subsections explore different filtration media used by nonwovens.

#### 3.1.1. Masks

Nonwoven fabric is a primary material used in the manufacturing of medical surgical masks and respirators. These masks are made by stacking together three layers of nonwoven materials [91]. Masks typically consist of an outer layer of hydrophobic spunbond fabric, an innermost layer of soft absorbent spunbond fabric, and a middle, sandwich layer of meltblown fabric. The innermost layer absorbs the moisture from the face of the bearer while the outermost layer is a waterproof nonwoven fabric, to prevent any external liquids being absorbed to the mask hindering the filtration of particles. The sandwiched middle layer is the filter layer which captures all airborne particles and provides protection [91,92]. Composite nonwovens are used to enhance the mechanical integrity of the high efficiency filtering layers. Due to the intrinsic properties of nonwovens, such as their lightweight and the physical barrier property with sufficient breathability, they are readily used in masks [91,92,93,94].

Meltblown fabrics are highly in demand for filtration-based applications. Most meltblown fabrics are fabricated with polypropylene (PP), polyethylene (PP), polyester (PET), nylon (PA6 or PA66), and polylactic acid (PLA). Meltblown polypropylene is conventionally used for surgical masks and is widely available in sheet form with trifold pleats [95]. Recently, electrospun filters have emerged as filter materials due to their higher filtration efficiency owing to the ultrafine nanofibers, especially for protection against small viral and/or bacterial particulates of 10–100s of microns [96].

#### 3.1.2. Respirators

**Figure 9:**
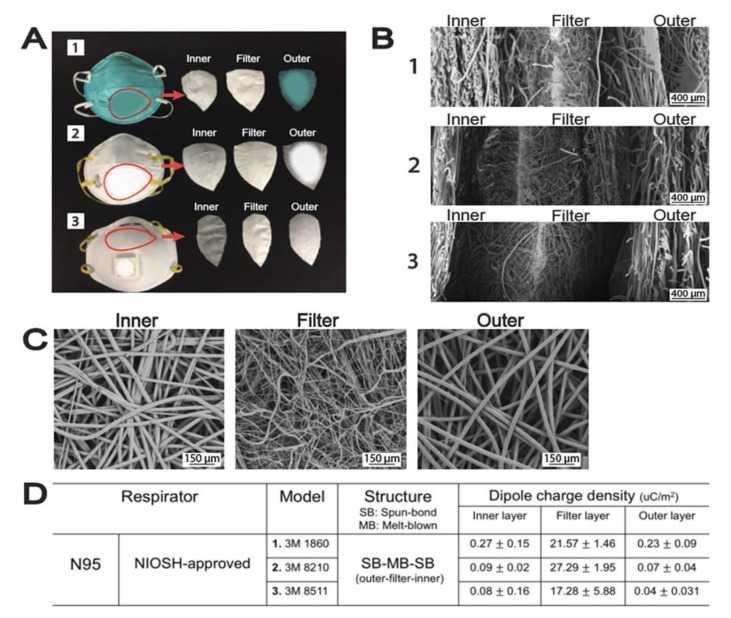
Overview N95–nonwoven layers and composite assembly. (**A**) Filter layers analyzed, (**B**) N95 layers: inner, filter and outer layers, (**C**) Comparison of pore size of the different layers, (**D**) Dipole charges on the filter layer for electrostatic filtration [107].

Respirators are made to protect effectively by filtering the air and fitting tightly to the face, while preventing the transmission of filtered matters to others. N95 masks are the most well-known facepiece respirator, of which the middle filter layer is meltblown polypropylene (PP) and acts as the primary filtering medium, as illustrated in Figure 9. The meltblown PP fabrics are electrostatically charged to attract and capture particulate matters of ~0.3 µm diameter; by NIOSH standard, N95 masks prevent 95% of particles >0.3 µm while staying well below the 350 Pa pressure drop [97]. They were originally designed to protect workers in the industrial and manufacturing environment to protect them from inhaling harmful particles that induce severe lung damage [97]. The N95 respirators were adopted in medical procedures to prevent blood splatters and pathogens by healthcare professionals [60,95,96,97,98]. During the pandemic, surgical masks and N95 were extensively used for protection from COVID-19 by the frontline workers and the public. There was a huge demand for these nonwoven respirators; although N95 dominated the market, emergent masks/respirators based on electrospun filters were also introduced to effectively filter out COVID-19 particulates [91,98]. Electrospun nanofibers are at least an order of magnitude smaller in mean diameter than meltblown, as such, nanofibers yield enhanced filtration efficiency as surface interaction is the major driving force in air filtration [99,100]. While a smaller fiber diameter, and hence a smaller pore size, yields less breathability than N95 [99], nanofiber-based respirators can retain filtration efficiency after washing as their primary filtration mechanism does not rely on electrostatic charges [101,102,103]. Their lightweight nature and uniformity and the consistency of the materials are also notable advantages over N95 [99,104]. Despite these advantages, electrospinning faces issues with scalability when compared with meltblown; the production rate increases linearly with the number of spinnerets [105,106].

#### 3.1.3. Antimicrobial Coating

Conventional surgical masks consist of a meltblown filter and spunbond inner and outer layers, which typically have a larger pore size (10–100’s of µm) than pathogens, as such, filters alone do not provide sufficient protection [92,108]. To increase the antimicrobial/viral efficacy of the nonwoven filters, antibacterial and/or antiviral coating is applied. Typically, surface treatment of the fabric is necessary to promote adhesion, then the antimicrobial coating is conformally applied to the nonwoven substrate [91,92,109]. The antimicrobial moieties, utilizing a high surface area of nonwovens, can then effectively kill microbial particles [91,92,109]. Desirable antimicrobial traits are the effective inhibition against a broad spectrum of viruses, non-toxicity to human, compatibility with resident skin microbiota, and being allergen-free, while not interfering with the textile quality of the PPE substrate [92,108,109,110]. Agents with different antimicrobial mechanisms can be applied at the same time via direct disinfection, receptor inactivation, and indirect disinfection [91,92]. These functionalized/coated PPEs aided in the suppression of viral pandemics such as COVID-19 [108,109,110]. Predominantly used agents are triclosan, quaternary ammonium compound, polyhexamethylene biguanides, N-helamine, N-Chloramine, hexachloropene, silver compound, natural bioactive compounds, antimicrobial acrylic fibers, copper-impregnated antimicrobial textiles, metals such as silver nitrate, mercuric chloride and tin chloride, silver nanoparticles, zinc oxide nanoparticles, and titanium dioxide nanoparticles [111].

#### 3.1.4. Others

Nonwoven filters are found in other applications, such as automotives. Predominantly, meltblown, carded, needle-punched PPs are used in cabin air filters, seat trims, vinyl roof and vinyl backing for seat covers, floor mats, wire insulations, fuel cells, door trims, reinforced tires, trunk liners, and wheel arch barriers. The primary functions of these nonwovens are for additional support, their lightweight, protection from wear and tear, their cost-effectiveness, their water resistance, and floor insulation from dust and noise [52,59,112]. While not strictly PPEs, these nonwovens do play a role in the protection of the driver. There are also nonwoven air filters used in automation to promote the respiratory protection of the drivers. For instance, nanofibers of 200–300 nm diameter are assembled on top of the pleated dust collection and engine air-intake filter substrate [95]. These nanofiber filters essentially form a “labyrinth” of fibers to filter the particles in the incoming air stream [95]. Other filter mediums are found in vacuum cleaners to effectively remove all the finer particles, allergens, and polluted air. Like N95, the filter media is made of electrostatically charged fibers called “electrets”, which are carded and needle-bonded nonwovens that create a high level of friction to acquire the electrostatic charges. Due to their electronegativity, this results in excellent filtration efficiency [60,94].

In food and beverages, nonwoven filters are used for processing and purifying potable water for safe consumption and are used in several industrial manufacturing processes. Rather than investing in expensive membrane systems and water treatments, these ultrafine nonwoven filters offer a cheaper alternative to reduce bulk contaminants, organics, and bacteria [60]. The chemical and bacterial resistance of the polyester media makes these filters fit for potable water, and industrial and well water applications [60]. In the chemical industry, filter papers that are absorbent, porous, nonwoven fiber materials are widely used in the manufacturing process [61]. In oil refineries, oil filters are used as oil does not wear out, it only becomes contaminated, leading to hydraulic failures due to the unclean oil. The oil is filtered down to 1 µm using the nonwoven filters, to prolong the oil and increase productivity [62]. In coal fired power stations, burnt coal is released as ash emission which must be prevented from passing to the atmosphere. Needle-punched fabric structures could lessen these emissions, causing high air flows and extended life [63].

### 3.2. Medical

Nonwoven fabrics are widely used as PPE in the medical sector. In the medical field, nonwoven garments, wipes, and ancillary fabrics are attractive for their sufficient mechanical protection and excellent viral/bacterial barrier properties, while their disposable nature renders it easy to stay sanitary [52]. The following subsections review various PPEs, both wearables and non-wearables, used in the medical field.

#### 3.2.1. Medical Garment

Surgical gowns and drapes are essential to protect the patient from infections, as well as the wearer from encountering bodily fluids and blood. Single use, nonwoven gowns and drapes are highly desirable compared with reusables as healthcare workers find it more sanitary [19,64]. The most highly used nonwoven fabrics for surgical gowns are spunbond, wet-laid, and “SMS”—a spunbond–meltblown–spunbond composite. The fibers are manufactured from wood pulp, cotton, polyester, and polyolefins [65]. Polypropylene nonwoven surgical gowns are available at 25–50 g/m^2^, while lightweight gowns are manufactured using a polyethylene (PE) coating over PP spunbond fabric of 30 g/m^2^. Disposable SMS gowns are manufactured in the range of 40–50 g/m^2^ [64,66,111]. The general requirements of these drapes and gowns include liquid repellency, antibacterial properties, comfort, tactile softness, strength, abrasion resistance, flame resistance, static safety, and toxicity [19,65]. For these surgical drapes, optimal fabric stiffness is critical to strike a balance between barrier performance and comfort.

Typical surgical gowns consist of three-layer composites: an outer, middle, and inner layer, as listed in Table 2. The outer layer is a spunbond fabric which acts as a mechanical barrier and a liquid repellent layer, while the two middle layers are meltblown fabrics used to mitigate moisture and filter bacteria. Finally, the innermost layer is a spunbond used for textile comfort as well as a mechanical barrier [19,65,66,111]. Similarly, for the respirator, plasma treatment can provide an improved barrier against blood and microbes [19,66,111]. Fabrics can also be treated with antibiotics and/or fluorochemical for viral/liquid control. For the medical sector, disposable gowns are beneficial as the contaminants are eliminated by proper disposal. About 15% of surgical infections are nosocomial and the use of surgical gowns and drapes plays a key role in protection [64,66]. Surgical wear for other body parts, such as drapes, caps, headcovers, and shoe covers, follows the same principle of layered assembly with additional functionalities provided by the coating [19,64,66,91,111].

#### 3.2.2. Wound Dressing and Ancillary Fabrics

Wound dressings are assembled with nonwoven layers that are highly absorbent, skin compatible, and air and moisture permeable, while striking the optimum stiffness in the whole dressing [19,111]. The main purpose of the dressing is to protect the wound from micro-organisms while promoting the absorption and distribution of exudates; other desirable traits include non-adherence, non-allergic, biocompatibility, cost effectiveness, and to allow sufficient gaseous and fluid permeability [64,111]. The nonwoven composite layers (Table 2) of the dressing consist of meltblown PP as a contact layer, spunlace cotton or viscose as the absorbent layers, and support material provided by a spunbond. Wound contact layers work as non-adherents with blood clotting and are responsible for the diffusion of the antibacterial component [64,111]. The first layer is a perforated film, while the secondary layer exudates absorption and distribution. Hydroentanglement, needling, and ultrasonic welding are used to bond three or more layers into a fabric [111]. Acrylic adhesive is coated over the base material, which gives structural stability to the dressing; a moist environment around the site prevents the additional loss of tissue and promotes the natural process of dermal and epidermal tissue generation [9,64,67,111].

Foam dressings have a perforated silicone wound contact layer, an absorbent core that consists of a thin sheet of polyurethane foam, and a piece of nonwoven fabric with a layer of superabsorbent polyacrylate fibers [19,64,68,69,111]. Vilmed nonwovens for wound pads are thermally bonded and used as backings for wound dressings and plaster strips. Orthopaedic cast paddings, which have optimum stretching properties, prevent constriction and provide air permeability for tissue growth under the dressings [19,64]. Chitin nonwoven dressings are produced by a special wet-laid process and have 3D structures which are ideal for extensive burns, scalds, and other traumas [19,64]. They inhibit bacterial growth, while stimulating new skin cell growth due to their excellent biocompatibility, leading to accelerated wound healing [19]. Silver dressing nonwoven fabrics, which are some of the most long-lasting dressings, exhibit antimicrobial properties for 7 days; the purpose of antimicrobials in these dressings is to prevent infection and to promote healing by tissue regeneration [64,67,111].

For post-surgical protection, different nonwoven products are required to offer necessary protection to patients. Underpads are needed as a protection for post-surgery bodily fluids that may be absorbed by the bed sheets and infect the patients. These underpads are made of a highly absorbent, moisture wicking surface on one end, while an impermeable layer is on the other end to prevent fluids soaking the mattress. Spunbond PP and SMS composite structures are extensively used in underpads [19,64,70]. Lightweight gauze products are needed mid- and post-surgery. These consist of an open web structure formed via spunlace. They typically consist of 70% viscose rayon and 30% polyester, which has high absorbency and low lint properties [64]. Lastly, wadding is a protection essential to prevent wound adhesion or fiber loss post-surgery. Orthopedic cushion bandages are used under plaster casts and compression bandages for padding and comfort [64]. These bandages are made of lightly needle-punched viscose to maintain lofty bulk volume, while providing cushion support. These are either fiber-bonded materials or spray-bonded with acrylics which weigh about 70 g/m^2^ [64].

Nonwoven medical wipes primarily entail disinfecting and incontinence wipes specifically designed for use in the healthcare sector. They exhibit good liquid absorbency, and are antimicrobial and lightweight, as with many other nonwovens. Typically, the wipes are manufactured across most production methods, i.e., air-laid, dry-laid, needle-punched, wet-laid, and spun-laid, which are all suitable for chemical impregnation with disinfectant [24,71]. The disinfectant wipes are typically used across many areas in the hospital, ranging from clean rooms, controlled-environment cleaning, and heavy- and light-duty cleaning.

### 3.3. Protective Garments

Protective garments encompass offering, at a minimum, physical protection to workers in the military, firefighting services, and law enforcement. Unlike the medical application and filters, the mechanical demands of these garments are quite high and often offer durability under more extreme temperatures and humidity. Often, these personnel also require additional protection such as chemical and/or biological protection, on top of the textile requirements. As such, nonwoven incorporation is more challenging as the garment needs to have all the properties essential for field use [8,69]. The following subsections examine the role of nonwovens in protective garments for firefighters and military personnel.

#### 3.3.1. Firefighters

**Figure 10:**
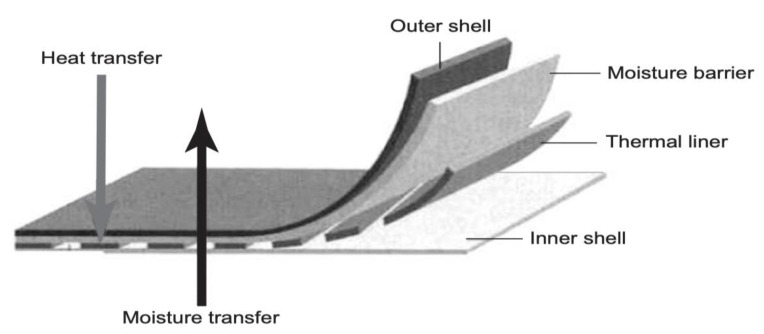
Structure of protective garment—fire fighters [69].

Protective garments for firefighters typically consist of four layers: an outer and an inner shell, a moisture barrier, and a thermal liner (Figure 10). These layers are collectively expected to provide heat, flame, liquid, chemical, and mechanical protection [8,69,72]. Hydroentangled aramids are well established moisture barrier substrates in firefighting garments [8]. These economical fabrics provide high temperature resistance, extreme durability, high tensile strength, water resistance, breathability, softness, and drape. These nonwovens are often used as linings within woven shell fabrics, which act as the primary mechanical barrier [8,72]. Many nonwoven manufacturers focus on manufacturing the latest technologies embedded in protective garments for firefighters that ameliorate the interaction between the worker and their garment. Among them, CRYON technology combines the comfort of natural cotton with the protection of modacrylic fibers and workwear using high-performance fibers such as Nomex, Kevlar, and poly (benzimidazole) PBI; these act as safer alternatives for flame resistant fabrics [69,72,73,74]. These suits are designed to allow for the better evaporation of sweat and are well ventilated to reduce the risk of burns. A firefighter suit includes coats, pants, underwear, helmets, boots, gloves, station wear uniforms, and breathing apparatus [74,75,76,77].

#### 3.3.2. Military Applications

Body armor is worn by the army and law enforcement personnel to primarily protect against gunfire or high velocity fragments. Modern body armor is categorized into two types: hard body armor and soft body armor [78]. Hard body armor, as the name implies, is made from rigid materials such as ceramics, reinforced plastics, metals, and composites; its primary function is to deflect bullets or high-velocity fragments on impact [78]. Soft armor is more flexible, lightweight and is composed of yarns with a higher specific strength than steel. Soft armor is made of fabrics with a high tensile strength, such as Kevlar, Spectra, Twaron, etc. Often, combining both the hard and soft armor maximizes protection as per the demands [79,80,81,82]. A combination of both the hard and soft armor as multi-layered protection gear can offer maximum safety. Yarns made of spun carbon nanotubes could be used in bulletproof vests, as they have large impact resistance.

While most traditional soft armors are woven yarns, instances of nonwovens are found as well [78]. Needle-punching can be advantageous in this operation due to its simplicity when compared with weaving; it was found that a needle-punched fabric with ballistic-resistant nylon could weigh just one third of a woven fabric while retaining 80% of its ballistic protection properties [78]. Another field study in 2002 has shown that the explosion fragment protection of needle-punched nonwoven fabrics was superior to woven aramid and PBO fabrics [78]. A hybrid combination of woven and nonwoven can be considered to synergistically protect against a variety of threats.

Bomb suits, unlike body armor, need to provide protection for the entire body and may be without gloves for maximum mobility and ease of use [78]. Mechanical bonding technologies are used in developing such military products with specific properties [80,81,82]. They offer thermal insulation to protect in extreme cold, water resistance to not allow the penetration of water or ice into the suits, and breathability to withstand excess heat. Multilayered needle-punched fabrics are used to incorporate filters and chemical protective suit liners, which can be functionalized using biocidal materials to enhance chemical/biological protection [77,78,79,80,81,82]. Since protection against chemical warfare agents (CWA) are critical for field use, sorption agents are incorporated within the liner along with a nonwoven mesh with adhesives. Activated carbon spheres are readily used as CWA-mitigant agents [83], although the advancement of other agents, such as a metal organic framework (MOF), functionalized porous silica, and their textile incorporation as liner materials are being considered as well [84,85].

### 3.4. Emerging Applications

The emergence of new materials (e.g., graphene, 1D and 2D nanomaterials) and manufacturing advancements (smart textiles [113,114]) have led to new nonwoven-based PPEs for masks, medical use, and protection. Table 3 below summarizes notable emerging nonwoven PPE applications in each field.

In general, the emerging applications entail incorporating additional functionality with minimal disruption to the base functionality of the current state-of-the-art. These functionalities entail “smart” sensor and actuator capabilities or provide additional protections. The development of the protection against multiple threats is especially critical for the protection sector, though it does not necessarily pertain to nonwovens alone. Future military PPE should concentrate on the development of full protection against multiple injuries, enhanced stealth, and complete coverage, without making it bulkier to wear and carry [119]. The recent development of miniaturized sensors and technologies may enable the reliable monitoring of individual wearers while tracking their physiological conditions [119].

## 4. Challenges and Outlook

Nonwovens are widely regarded as disposable, often meant for one-time use. On the one hand, single-use nonwovens are attractive for certain applications for the purpose of being sanitary at an affordable price, e.g., filtration and medical applications as discussed. On the other hand, two major issues have emerged due to their single-use nature: the supply chain and global waste. In the early days of the COVID-19 pandemic, the nonwoven industry struggled to meet the sudden surge of demand for PPE across the globe; this impacted end-use industries which were receiving a regular supply of nonwovens, i.e., automobiles, wipes, tissues. Some sectors saw more immediate impacts, such shortages in wipes and tissues, resulting in a hike in price. Other sectors saw more delayed effects, such as automobiles being sold at higher prices with longer lead times. While changes in manufacturing policies diverted some of the supply chain issue for PPEs, these served as a stop-gap measure to divert immediate crisis.

The other major issue is the environmental impact of single-use nonwovens. For example, due to the recent COVID-19 pandemic, polymer-based PPE waste has significantly increased [86]. China’s medical-related waste has increased six-fold when compared with pre-COVID-19, to nearly 240 tons of waste per day [87]. Synthetic polymers, e.g., polyester or polyolefins, which are predominantly used in nonwoven PPE, also add to the sustainability issue. Due to their non-biodegradability, these products are incinerated, which generates further carbon emissions or landfills, where they can persist for ~450 years [91]. During the pandemic, approximately 44 million PPE nonwoven products were used daily, predominantly those made of polypropylene, leading to nearly 15,000 tons of waste every 24 h worldwide [91]. Although nearly 95% of these wastes are, in principle, recyclable, about 85% of them end up in landfills; some of this can be attributed to policy, public awareness, and mixed waste streams [88].

**Figure 11:**
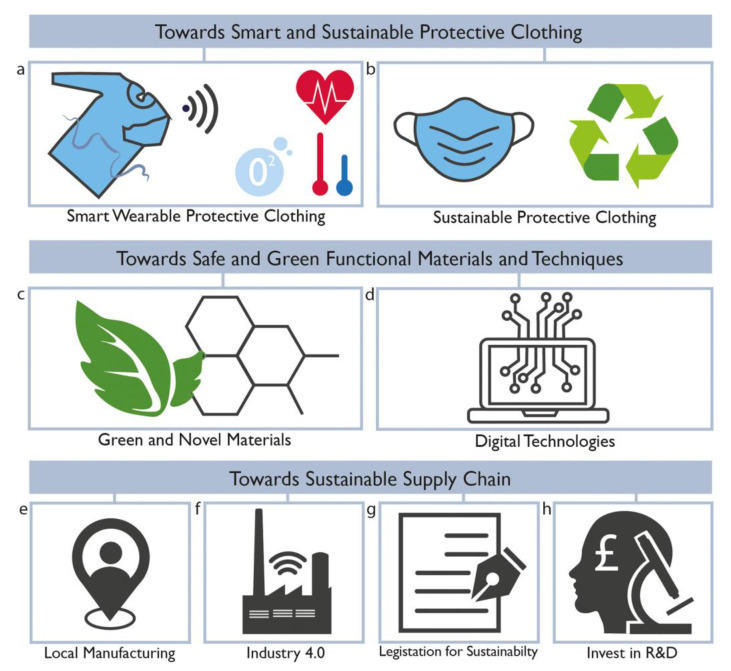
Future research directions and recommendations. (**a**) Smart wearable protective clothing to monitor a wearer’s body vitals such as temperature, heart rate, and oxygen saturation level. (**b**) Sustainable protective clothing: reusable, washable, and recyclable. (**c**) Green, natural, and novel materials for functional finishes on textiles. (**d**) Integrate digital technologies for processing personal protective clothing. (**e**) Local manufacturing of personal protective clothing for healthcare applications. (**f**) Industry 4.0 for manufacturing of protective clothing. (**g**) Government legislation for using sustainable PPE. (**h**) Public and private funding in R&D to develop new and innovative technologies [120].

Going forward, material and process innovations are needed to meet these challenges. The enhanced functionality and durability of the nonwovens can lead to the increased lifetime of the fabric thus decreasing the accumulation of waste. Figure 11 summarizes recommended future directions to address the sustainability issues stemming from nonwoven PPE; the authors point to the review article [120] for more insights. One such innovation is the incorporation of shear-thickening fluid (e.g., silica nanoparticles dispersed in polyethylene glycol (PEG)) to further enhance the impact and ballistic protection [89,90]. Such improvements can render the use of PPE for longer periods of time, leading to more efficient use of the materials used and possibly less production required. Other approaches entail high-value additive functionality, such as electronic textiles. If the PPE serves its role beyond textile and barrier functionality, it is more likely to be retained for multiple uses rather than remaining single use. For the medical sector, e-textile technologies can be integrated with the PPE to constantly check body temperature and respiratory activity to monitor the patient’s health more closely [91,120]. Innovations on the components of e-textiles can also have long-lasting sustainability effects. Two-dimensional materials, such as graphene, are known for their outstanding electrical and mechanical properties and have drawn significant interest in the field of wearable e-textiles. Graphene-based e-textiles are more reusable, washable, durable, and potentially more environmentally friendly compared with the existing metal-based e-textiles [91]. One good example of this is a reduced graphene oxide-coated cotton spunlace nonwoven fabric [89,90]. In the case of protection suits, efforts to develop thinner and more flexible batteries to reduce the weight burden and their usability address the issues of smart protective clothing and PPE [90]. For chemical/biological PPEs, one can identify the optimal point of disposal due to contamination. For example, the integration of sensors to indicate whether the suit has reached above the safe threshold of contaminant concentration would encourage smarter and more efficient disposal [121]. Proper decontamination procedures, such as UV-irradiation, may also help extend the lifetime of PPE [122,123].

Another strategy is to use recyclable or renewable polymer sources to manufacture nonwovens. A recent study showed that low melting temperature bicomponent polyester fibers were developed, where poly (hexamethylene terephthalate) (PHT) shells were co-extruded with a poly (butylene terephthalate) (PBT) core via melt spinning [124]. These fibers were then thermally bonded as nonwovens; the fibers could also be chemically recycled via methanolysis [124]. Another example is incorporating enzyme-receptive compounds with commodity thermoplastics to depolymerize the polymer [125]. Other cases of upcycling end-of-life products into nonwoven PPE have been found; we point the readers to a highly relevant review article [90].

Integration across various disciplines is crucial to holistically innovate “smart” PPEs, i.e., more sustainable, multifunctional PPEs without compromising baseline comfort and breathability [89]. While the material innovation and smart functionalities inevitably come at the increased cost of the final product, large scale production that encompasses all functionalities, driven by universities (fundamental innovation), industry (applied innovation and manufacturing), and governments (policy) may accelerate more economical production.

## 5. Conclusions

Nonwovens are widely used in multiple sectors including manufacturing, medical, military, law enforcement, and fire fighter workspaces. From conventional fabrics to nonwovens, the market is rapidly changing, hence the landscape of their applications and global impact; the consequences of the accelerated changes due to the recent COVID-19 pandemic are felt to this day. They are lightweight, breathable, single-use disposables, with high surface areas available to functionalize, which is ideal for PPE applications. Owing to fabrication and bonding processes, the processing–structure–property relationship dictates their morphology and end-use, e.g., water repellence and filtration change with respect to the diameter and pore sizes. Owing to the emergence of nanofiber spinning, the efficiency of the filters and their reusability are improved over the single-use state-of-the-art. The use of nonwovens is in every field, from respirators for industrial use, masks for healthcare workers and the public, in automobiles, and in protective garments. Sustainability issues stemming from the single-use nature of nonwoven PPE need to be addressed, and this should be led by material and processing developments. High-value additives or functionalities are desirable to increase the lifetime of these PPEs, while other solutions, such as reusability, renewable sources, and recycling, are also important.

## Figures and Tables

**Table 1:** Polymer-laid spinning techniques and their fiber dimensions, advantages and disadvantages, and applications (adopted and modified from [49].).

Methods	Fiber Dimensions	Advantages	Disadvantages	Applications
Spunbonded	Microfibers	Randomly oriented fibers offer good mechanical strength; mass production; good thermal properties; permeability; high tear strength; abrasive resistance	Lesser filtration efficiency due to larger fibers although reducing fiber diameters can achieve improved protection properties and can be used for other applications; poor barrier property	Medical and healthcare; construction; agricultural packaging; protective materials; filtration—due to low filtration efficiency (can achieve only 88.27%) it can be used as outer/inner mask layers
Meltblown	Micro/nanofibers	Does not require solvents leading to no pollution; high barrier property; mass production; wide range of polymers	Filtration can only be met using electrostatic electret; wear resistance and mechanical properties are lower but better than electrospun/centrifugal spun fabrics; larger diameter; higher temperature air is required	Filtration—air, liquid, oil/water separation; medical protection—PPE, masks
Electrospinning	Nanofibers	High barrier property; simple device; ultrafine fiber diameters; wide range of polymers and polymer composites	Safety issue—high voltage, poor mechanical strength compared with spunbond, and meltblown; low productivity; better spinning stability needed for large industrial scale production	Biomedicine; filtration materials; protective clothing
Centrifugal spinning	Nanofibers	Both conductive and non-conductive polymers; high barrier property; safer to use compared with electrospinning; when polymers are melt centrifugal spun, can be solvent free causing no pollution.	Poor mechanical strength compared with meltblown and spunbond; complex machinery for lab-scale; need to optimize spinning concentration to attain desired fiber sizes with good properties; less homogeneous fibers compared with electrospinning; higher speed and performances need advanced motor and bearings; melt centrifugal spun has fibers in microns	Biomedical and tissue engineering-based applications such as wound dressings, etc.; filtration; sensors
Solution blow spinning	Nanofibers	High barrier property; high voltage is not needed; wide range of polymers; ultrafine fiber diameters; non-toxic solvents used for spinning; thermal degradation of polymers can be avoided	Poor mechanical strength compared with spunbond and meltblown; unintentional fiber entanglement	High temperature thermal insulation; air filtration; water treatment; electronic devices; biomedical applications
Flash spinning	Micro/nanofibers	Good barrier property; excellent tear and puncture resistance; water resistance and mechanical strength; good permeability; can use insoluble polymers to prepare fibers	Unintentional fiber entanglement	Air filtration; medical protective materials

**Table 2:** Nonwoven PPE applications, process, structures [19,55,56,57,58,59,60,61,62,63,64,65,66,67,68,69,69,70,71,72,73,74,75,76,77,78,79,80,81,82,83,84,85,86,87,88,89,90].

Applications	Products	Method Used	Layers
Filtration	Masks	Spunbond, meltblown, electrospun	*Outer*: spunbond *Middle*: meltblown or electrospun *Inner*: spunbond
	Respirators	Meltblown, electrospun	*Outer*: meltblown *Middle* (*filter*): meltblown or electrospun *Inner*: meltblown *Support layer*: modacrylic
Medical	Surgical gowns	SMS, wet-laid, spunbond	*Outer*: spunbond repellent fabric, *Middle layers*: meltblown for fluid control and bacterial layer *Inner*: spunbond
	Wound dressings	Spunlace, needle-punched, hydroentanglement	*Outer*: protective backing antibacterial layer *Middle*: spunlace nonwoven, sandwiched between super absorbent and absorption Polyurethane foam, *Inner*: wound contact silicone layer with adhesive
	Underpads	Spunbond, SMS	*Outer*: spunbond backing layer *Middle*: SMS *Inner*: spunbond
	Medical gauze	Spunlace, ultrasonic bonded	*Outer*: spunlace *Middle*: spunlace *Inner*: ultrasonic bonded with a textile mesh
	Wadding	Needle-punched, meltblown	Composite layers of needle-punched encapsulated in meltblown
	Medical clothing	SMS, spunbond, hydroentanglement, wet-laid	*Outer*: SMS *Middle*: meltblown *Inner*: SMS
Protection	Firefighter’s garments	Hydroentanglement	*Outer*: a shell fabric *Middle*: moisture barrier, thermal barrier *Inner*: shell fabric
	Military garments	Hydroentanglement, needle-punched	Outer shell, inner shell, middle-moisture barrier, thermal barrier layers

**Table 3:** Emerging applications of nonwoven PPEs categorized by field.

Field	Applications
General PPE	Graphene-modified personal protective clothing [115]
Mask/ Respirators	An earbud incorporated into masks for better quality calls. Self-disinfecting face masks with two conductive fabrics sandwiched by a dielectric layer [116].
	Breath-activated antibacterial face mask using Ag/cotton/Zn nonwoven to generate a microelectronic field. Excellent electroactive antibacterial activity against E. coli and S. aureus [117].
Medical	Cellulose fiber-based hierarchical filter materials for protective clothing [118]
	Reusable and bio-based medical protective fibers [49]
	Antiviral/antimicrobial medical protective fibers [49]
	Visual detecting medical protective fibers [49]
Protection	Intelligent thermostatic garments, i.e., suits for thermoregulation, microclimate regulation systems, water storage, waterproof, breathable, thermoregulation, release control carrier for functional molecules, camouflage uniforms—stealth and protection by material color change to blend with external surroundings [119]
	“Warrior Web” using Bionic technology, intelligent combat clothing, to protect health and safety of soldiers [119]
	Nuclear, biological, chemical (NBC) protective clothing [119]

## Data Availability

Not applicable.
